# Titanium Porous Coating Using 3D Direct Energy Deposition (DED) Printing for Cementless TKA Implants: Does It Induce Chronic Inflammation?

**DOI:** 10.3390/ma13020472

**Published:** 2020-01-19

**Authors:** Dong Jin Ryu, Chung-Hee Sonn, Da Hee Hong, Kyeu Back Kwon, Sang Jun Park, Hun Yeong Ban, Tae Yang Kwak, Dohyung Lim, Joon Ho Wang

**Affiliations:** 1 Department of Orthopedic Surgery, Samsung Medical Center, Sungkyunkwan University School of Medicine, Seoul 06351, Korea; mdryu24@naver.com (D.J.R.); kwonkyubaek@naver.com (K.B.K.); princepsj@hanmail.net (S.J.P.); 2 Samsung Biomedical Research Institute, Samsung Medical Center, Sungkyunkwan University School of Medicine, Seoul 06351, Korea; chsonn@hanmail.net (C.-H.S.); dahee1994@hanmail.net (D.H.H.); 3 Department of Mechanical Engineering, Sejong University, Seoul 05006, Korea; ban2946@sejong.ac.kr (H.Y.B.); rhkrxodid@sejong.ac.kr (T.Y.K.); 4 Department of Health Sciences and Technology, SAIHST, Sungkyunkwan University, Seoul 06351, Korea; 5 Department of Medical Device Management and Research, SAIHST, Sungkyunkwan University, Seoul 06351, Korea

**Keywords:** direct energy deposition, titanium porous coating, cementless TKA, 3D printing, chronic inflammation

## Abstract

Because of the recent technological advances, the cementless total knee arthroplasty (TKA) implant showed satisfactory implant survival rate. Newly developed 3D printing direct energy deposition (DED) has superior resistance to abrasion as compared to traditional methods. However, there is still concern about the mechanical stability and the risk of osteolysis by the titanium (Ti) nanoparticles. Therefore, in this work, we investigated whether DED Ti-coated cobalt-chrome (CoCr) alloys induce chronic inflammation reactions through in vitro and in vivo models. We studied three types of implant surfaces (smooth, sand-blasted, and DED Ti-coated) to compare their inflammatory reaction. We conducted the in vitro effect of specimens using the cell counting kit-8 (CCK-8) assay and an inflammatory cytokine assay. Subsequently, in vivo analysis of the immune profiling, cytokine assay, and histomorphometric evaluation using C57BL/6 mice were performed. There were no significant differences in the CCK-8 assay, the cytokine assay, and the immune profiling assay. Moreover, there were no difference for semi-quantitative histomorphometry analysis at 4 and 8 weeks among the sham, smooth, and DED Ti-coated samples. These results suggest that DED Ti-coated printing technique do not induce chronic inflammation both in vitro and in vivo. It has biocompatibility for being used as a surface coating of TKA implant.

## 1. Introduction

Total knee arthroplasty (TKA) is a reliable procedure that is used in orthopedics to treat end-stage osteoarthritis in knee joints [1]. Over the past three decades, the survival rate of TKA has increased remarkably with surgical technique, implant design, and postoperative management. Regarding implant fixation, the most common method to secure fixation is the cementing technique [1,2]. Although cemented TKA is reported to have excellent long-term survival, cement has poor resistance to shear force and is associated with the risk of aseptic loosening of the tibial component. Osteolysis is known to be induced by small or nano sized particles produced from bone cement or metal debris and by the void (or interface membrane) formed at the interface between the bone and cement surface. Indeed, because of the discrepancy of the material property between the bone and cement, the interface membrane formation is inevitable after TKA surgery [3]. Thus, long-term longevity of the interface is compromised, especially in young or obese patients [4,5,6,7]. 

Cementless fixation was developed to achieve a more physiological fixation between bone-implant surface and to improve longevity. However, some reports of cementless TKA designs have since raised concerns regarding failure of fixation, early failure, and poor clinical outcomes [8,9,10,11]. Furthermore, the immediate fixation stability and the proved good survivorship of cemented TKA makes transitioning from this technique difficult for the majority of surgeons [12].

To improve osseointegration of bone-implant surface, cobalt–chromium (CoCr) alloys which have high strength, high corrosion resistance, flexibility, and good biocompatibility are used as base [13,14]. And the surface was created to porous structures by plasma sprays, fiber metals, and bead coatings [15,16]. As a result, the survival rate improved remarkably from the first generation model, and in recent years there have been reports of clinical results that are almost similar to that of the cemented implant [17,18,19]. Despite recent advances in mechanical engineering, there are still concerns about the mechanical stability and satisfactory outcomes of the cementless TKA implant.

Newly developed 3D printing direct energy deposition (DED) is a method for creating features via application of the additive through a laser using sprayed titanium (Ti) powder, rather than a bottom bed [14,20]. This method is inferior as compared to powder-bed-fusion (PBF) for the fabrication of complex features; however, extremely high mechanical strengths, similar to that of forged metal, make the additive on the surface of the complex feature suitable for knee implants [14,21,22,23].

There is previous in vivo study that evaluated the osseointegration of DED technology [14]. Of course, osseointegration is important, however before that, it is necessary to assess the incidences of inflammation. Despite the fact that CoCr and Ti are commonly used substances in implant manufacturing, there is a risk that the residual remains or Ti nanoparticles may occur because of wear. The osteolysis process can be described in three stages: (1) Exaggerated inflammation induced by macrophages and osteoclasts; (2) disruption of bone formation; and (3) disruption of bone regeneration as a consequence of the increased cytotoxic response [24]. The macrophages and neutrophils are contributing to an impaired inflammatory phase. In acute phase response signaling, interleukin (IL)-6 and IL-1β showed strong upregulation [25,26]. Subsequently, the GSK-3β/β-catenin signal pathway regulated osteogenic inhibition and bone destruction [27]. Even if early osseointegration is achieved, continuous inflammatory reaction may eventually result in osteolysis [25,28]. Here, we evaluated whether DED Ti-coated on CoCr alloy stimulate chronic inflammatory reaction both through in vitro and in vivo models.

## 2. Materials and Methods

### 2.1. Manufacturing of Specimens

**Figure 1:**
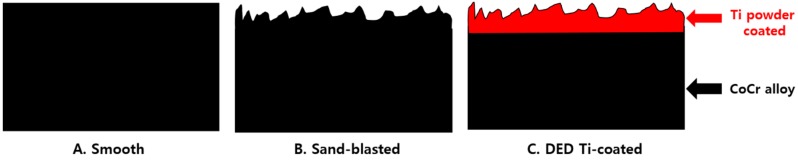
(**A**) schematic of a specimen used for experiments (**A**) smooth, (**B**) sand-blasted, (**C**) direct energy deposition (DED)-Ti coated (black color: cobalt chrome alloy, red color: Ti powder coating).

A porous layer of pure Ti (grade 2, ASTM F1580, Advanced Powders and Coatings, Inc. (AP&C), Boisbriand, QC, Canada) was coated on a CoCr substrate following the DED process. The porous structure was then manufactured using a 3-D computer-assisted design program that created a sufficient fixation force by matching the material to the properties of cancellous bone. The surface was irradiated as follows: laser power: 100 W; scan speed: 1.5 m/min; power delivery rate: 2.2 g/min by following a pre-programmed path along a grid, which formed a melted pool. Next, metal powders were sprayed and laminated onto the surface to create a coating layer (average thickness: 500 µm) [29,30]. In addition, smooth (polished) and sand-blasted specimens were prepared for comparison (Figure 1).

### 2.2. Sample Preparation and Test Groups

We studied three types of implant surfaces (smooth, sand-blasted, and DED) in vitro and in vivo to compare the inflammatory reaction using the Cell Counting Kit-8 (CCK-8)) assay, inflammatory cytokine assay, immune profiling, and histomorphometric analyses. The only media without cells were set to negative control to prove that the results were true positive and not due to chance in the in vitro study. To set the baseline by positive control, the cell only group was added in the in vitro experiments, and the sham group was added in the in vivo model. Three types of specimen (n = 54; diameter: 14.6 mm; height: 3 mm fitted for 24 well plate), namely smooth (n = 18), sand-blasted (n = 18), and DED Ti-coated (n = 18), were manufactured for in vitro studies. Similarly, for in vivo studies, three types of specimen discs (diameter: 6 mm; thickness: 3 mm) were manufactured (n = 36): (1) smooth (n = 12), (2) sand-blasted (n = 12), and (3) DED Ti-coated (n = 12). The specimens were rinsed three times with DNase/Rnase-free water and soaked in triple distilled water for 24 h and then washed again in double distilled water. Subsequently, they were dried completely and autoclaved at 121 °C for 15 min, pressure of 1 kg/cm^2^.

### 2.3. In Vitro Preparation

All cell culture biologics were purchased from Gibco BRL (Grand Island, NY, USA) and HyClone (GE Healthcare Life Sciences Korea, Seoul, KOREA). The CCK-8 Kit for cell viability assay was purchased from Dojindo Molecular Technologies (Rockville, MD, USA), flow cytometry kit from Abcam (Cambridge, UK), and LEGENDplex kit for cytokine assay was from BioLegend (San Diego, CA, USA).

#### 2.3.1. Viability Analysis on Culture of Fibroblasts

Mouse fibroblastic cells (L929; CCL-1; ATCC, Manassas, VA, USA) were cultured in Roswell Park Memorial Institute (RPMI 1640 Medium) supplemented with 10% fetal bovine serum (FBS), 100 IU/mL penicillin, and 100 µg/mL streptomycin (all from Gibco-BRL; Thermo Fisher Scientific, Inc., Waltham, MA, USA) under standard cell-culture conditions (37 °C, 95% air). Fibroblasts are usually not in direct contact with the implant surface, but have been widely accepted as one of the most sensitive cell lines for inflammatory reactions. Thus, we considered fibroblasts as an appropriate in vitro model to evaluate biocompatibility of DED specimens [31,32]. L929 cells were seeded in 24-well culture plates at a concentration of 5 × 10^3^ cells per well and were cultured on day 1, 3, 5, and 7. To assess the differences in early cell concentrations, low (3 × 10^3^ cells) and high (1.2 × 10^4^ cells) concentrations were re-examined on day 1, 3, and 5. The CCK-8 test was used for assessing cell viability. After incubation, the solution of CCK-8 (60 µL) was added to each well and mixed with the medium. Absorbance was then measured by a microplate reader at 450 nm wavelength.

#### 2.3.2. Inflammatory Multiplex Cytokine Assay

After 3 days of incubation (as detailed in Section 2.3.1), the supernatant from each well was taken for the cytokine assay. Reagents and standards were made according to the kit manual [33]. When the samples were ready for flow cytometry, FACS verse was used for flow cytometry and data was further analyzed by LEGENDplex v.8.0 software. Cytokines are small glycoproteins that act as signaling molecules and mediate communication between cells. They regulate inflammation and hematopoiesis. This assay allows simultaneous quantification of 13 mouse cytokines. We focused on six kinds of cytokines, namely TNF-α, MCP-1, IL-1β, IL-6, IL-12p70, and GM-CSF [34]. 

### 2.4. In Vivo Preparation

**Figure 2:**
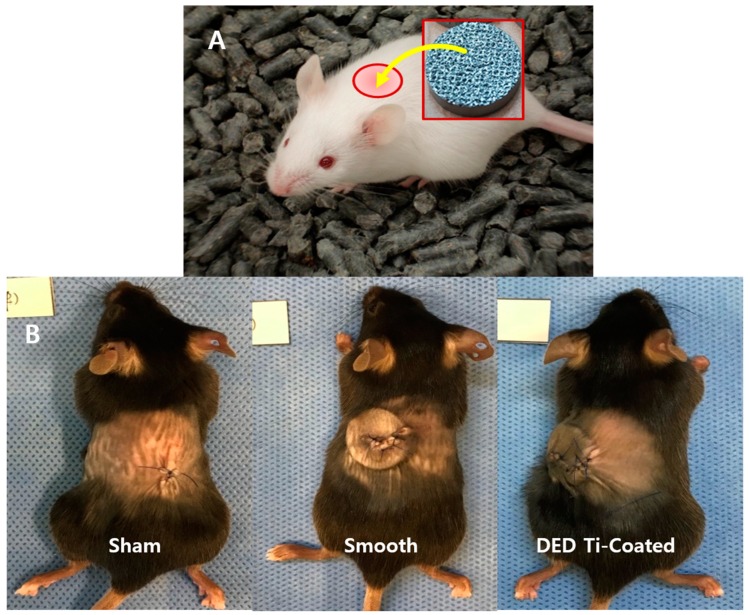
(**A**) The experimental specimen was inserted into the dorsal subcutaneous layer. (**B**) After inserting the specimen into the mouse. Specimens were not inserted in the sham group after incision as control group.

Thirty-six full-grown C57BL/6 mice (weight: 20 ± 0.8 g) were assigned as the experimental subjects. Animal management and surgical procedure were performed in accordance with the National Institutes of Health guide for the care and use of laboratory animals. We have received an approval for this study protocol by the Ethics Committee on Animal experimentation at Samsung Medical Center (SMC 2018-0713-002). General anesthesia was induced by intramuscular injection of ketamine 2 mg/kg and xyalazine 0.2 mg/kg. The mid-dorsal skin of each subject was shaved and sterilized with povidone-iodine. In the supine position, the mid-dorsal skin was incised transversely about 2 cm. The experimental specimen was inserted into dorsal subcutaneous layer and the wound was closed with nylon 5-0 (Figure 2).

Following implantation, approximately 700 μL of blood were taken from the heart on day 1, day 2, week 1, 2, 4, and 8 for use in the immune assay. The collected blood sample was centrifuged at 2500 rpm, for 20 min, at 4 °C condition and the serum was used for multiplex cytokine assays while the lower layer was used for immune profiling. At weeks 4 and 8, the mice were sacrificed using CO_2_ gas and samples were collected from the dorsal side skin to the subcutaneous tissue including experimental specimen en bloc [35]. 

#### 2.4.1. Immuno Profiling and Inflammatory Multiplex Cytokine Assay

The suspension was washed and adjusted to a concentration of 1 × 10^6^ cells/mL in ice cold phosphate buffered saline (PBS), 10% fetal calf serum (FCS), 1% sodium azide. Next the suspension was centrifuged at 400*g* for 5 min. Cells were stained in polystyrene round bottom 12 × 75 mm^2^ Falcon tubes. Subsequently, 1 μg/mL of conjugated primary antibody was added and incubated for 30 min at 4 °C. The samples were then washed three times by centrifugation at 400*g* for 5 min and re-suspended in 500 µL to 1 mL of cold PBS, 10% FCS, 1% sodium azide. Finally, the samples were kept at 4 °C until use according to manufacturer protocols. For inflammatory multiplex cytokine assay, the serum portion after centrifugation was used according to the aforementioned method.

#### 2.4.2. Tissue Histomorphometry

**Figure 3:**
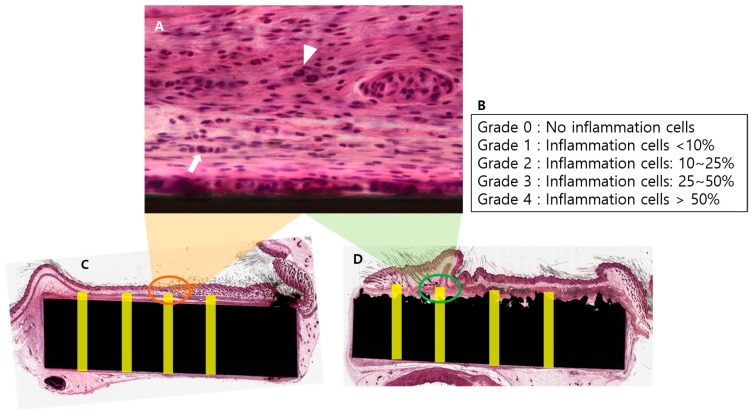
(**A**) The inflammatory cells including granulocytes (arrow heads) and monocytes (arrow) are observed in the area that we set. (**B**) The ratio of inflammatory cells to total cell in the set area was evaluated as grade using semi-quantitative grading system. (**C**, **D**) For each sample, four zones were set to be of 3.3 mm height and 0.5 mm width from the floor of the implant by selecting an area that is in well contact with the implant and the skin.

The harvested soft tissue was dehydrated with alcohol and soaked in Technovit 7200 resin (Heraeus Kulzer, Morphisto, Frankfurt, Germany). The tissues were embedded in paraffin for a light system (Exakt Technologies Inc., Oklahoma City, OK, USA). The block was then sliced into 50 μm-thick sections using a hard tissue slicer (Struers, Willich, Germany). These sections were then stained with hematoxylin and eosin (H&E; Sigma-Aldrich, St. Louis, MO, USA). Microscopy images were obtained using by X12.5, X100 (BX51, Olympus, Tokyo, Japan). For each sample, four zones were set to be 3.3 mm height and 0.5 mm width from the bottom of the implant. We tried to select the most representative zone within the images in terms of tissue density and maintenance of contact surface with implant and skin to avoid the bias. The zones from each implant were analyzed by semi-quantitative inflammatory cell grading system [36,37,38] (Figure 3). The score of 0 indicated no presence of inflammatory cells (including macrophage, monocyte, granulocyte, lymphocyte and giant cell) in the examined surface area; 1, the presence of inflammatory cell less than 10% of all cells in the examined surface area; 2, the presence of inflammatory cell in 10–25% of all cells in the examined surface area; 3, the presence of inflammatory cell in 25–50% of all cells in the examined surface area; 4, the presence of inflammatory cell more than 50% of all cells in the examined surface area. It was performed at a magnification of 100× by a well-trained researcher (DH Hong). 

### 2.5. Statistical Analysis

We compared the value of each test among the groups using a Kruskal–Wallis test. The level of statistical significance was set at *p* < 0.05. The Mann–Whitney U was used to compare between-group differences assessed using Bonferroni’s adjustment for multiple testing. Statistical analyses were performed using SPSS® 25.0 software (SPSS, Chicago, IL, USA).

## 3. Results 

### 3.1. In Vitro CCK-8 Assay

**Figure 4:**
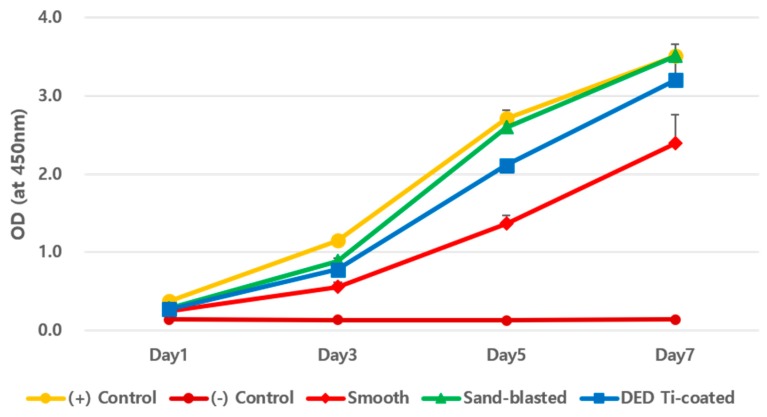
CCK-8 assay results for each specimen in a time dependent manner. The positive control, sand-blasted, and DED Ti-coated groups reached near their maximum value on day 7. However, the smooth group showed relatively low viability, but were not statistically significant (day 5 *P* = 0.1, Day 7 *P* = 0.1).

**Figure 5:**
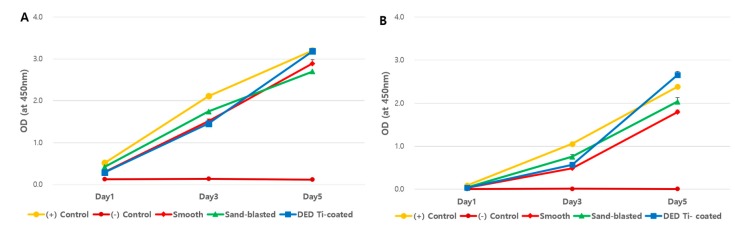
Results of re-testing using low ((**A**): 3 × 10^3^ cells) and high ((**B**): 1.2 × 10^4^ cells) concentrations of cells to determine the effects of initial cell concentration. All four experimental groups showed similar viability pattern and there were no statistically significant differences.

The cell viability on each specimen was checked by day 1, 3, 5, and 7. As shown in Figure 4, the time dependent increase in CCK-8 assay suggested that all of the surfaces revealed cytocompatibility without a prominent decrease in the detection values. DED Ti-coated specimens are also found to increase the number of cells without any statistically differences. In this study, cell viability was elevated in the DED group compared to the smooth group but this was not statistically significant (*p* = 0.1). A similar pattern of cell growth was revealed in repeated experiments to compare the results of different cell concentrations during 5 days among the four groups (Figure 5A,B).

### 3.2. In Vitro Inflammatory Cytokine Assay

**Figure 6:**
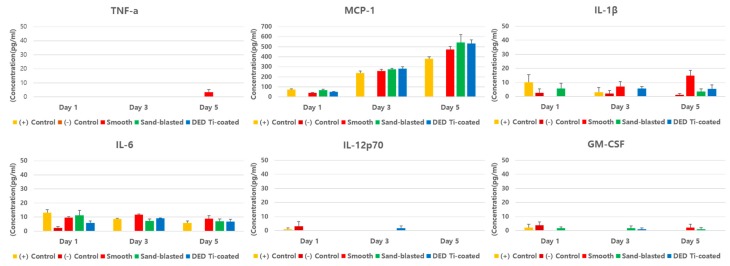
The results of the inflammatory cytokine multiplex assay assessment in vitro study. There were no statistically significant differences in the four experimental groups.

The results of the inflammatory cytokine multiplex assay assessment revealed no statistically significant differences in the four experimental groups (all *p* > 0.05, all of day 1,3, and 5, Figure 6).

### 3.3. In Vivo Immune Profiling

**Figure 7:**
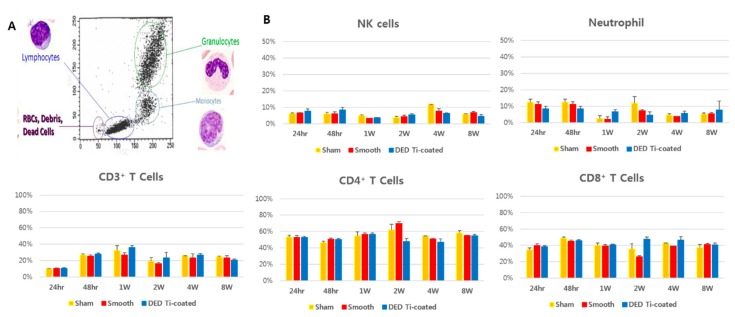
The results of in vivo immune profiling of blood samples. (**A**) Categorized into a specific group of inflammatory cells based on measurements according to the established criteria. (**B**) The results of the immune profiling assay. There were no statistically significant differences in all type of inflammatory cell among three experimental groups. The ratio of CD3^+^ T cell increased between 1 and 2 weeks, then decreased to normal levels. This increase was also measured in sham group without implanting specimens.

The cells that are responsible for immune responses to inflammation in the blood are neutrophils, CD3^+^ T cells, CD4^+^ T cells, CD8^+^ T cells, and NK cells [39]. Among them, the ratio of CD3^+^ T cell increased between weeks 1 and 2, then decreased to normal levels (Figure 7). This increase was also measured in the sham group which had no implanted specimens. Compared with the sham group, there were no differences in the experimental group (all *p* > 0.05). The observed increase was in response to the surgery itself, and the results showed that there were no specific inflammatory responses.

### 3.4. In Vivo Inflammatory Cytokine Assay

**Figure 8:**
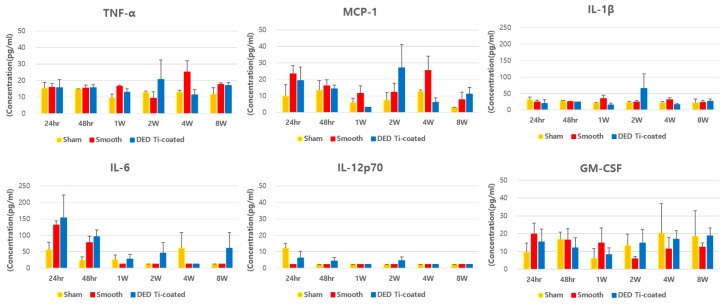
The results of the in vivo inflammatory cytokine multiplex assay of blood samples. There were no statistically significant differences in the three experimental groups.

None of the experimental groups showed statistically significant increases in the concentration of inflammatory cytokines compared to the sham group (all *p* > 0.05, Figure 8).

### 3.5. In Vivo Histomorphometry

**Figure 9:**
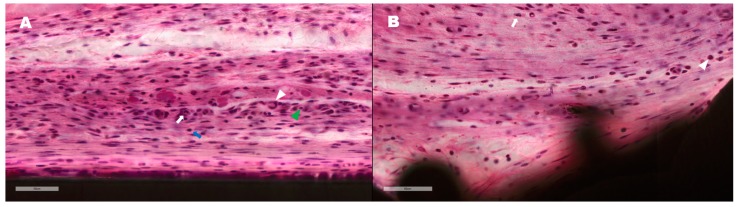

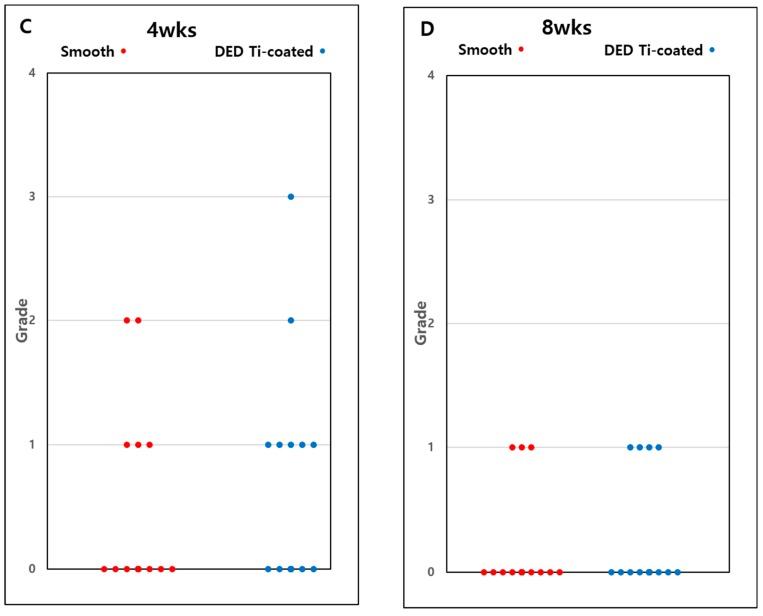
Evaluation of the degree of inflammatory cells by semi-Quantitative Grade System. Representative images of smooth (**A**) and DED Ti-coated (**B**) at 4 weeks after implantation. Macrophage (Green arrow head), lymphocyte (blue arrow), granulocytes (white arrow heads), and monocytes (white arrow) were observed in both specimens, however, not more than 10% of all cells. Both representing zone evaluated as grade 1. The result of semi-Quantitative Grade analysis represented at 4 weeks (**C**) and 8 weeks (**D**). The expression level of inflammatory cells was decreased at 8 weeks (**D**) compared with 4 weeks (**C**), however, there was no statistically significant difference (smooth group *P* = 0.413, DED Ti-coated group *P* = 0.219). There was no significant difference between the smooth and DED Ti-coated groups at 4 (*P* = 0.551) and 8 weeks (*P* = 0.755).

The semi-quantitative degree of inflammation evaluated by the presence of inflamed tissue involved with polymorphonuclear cells (mostly neutrophils) and lymphocytes. Some inflammatory cells were identified in parts of the smooth and DED-Ti coated specimens in a few sections of zones. However, there was no statistical difference in the grade of the inflammation between the groups at 4 weeks (*P* = 0.551) and 8 weeks (*P* = 0.755) (Figure 9). There were no statistically significant differences within each group (smooth group *P* = 0.413, DED Ti-coated group *P* = 0.219).

## 4. Discussion

The aim of the present study was to investigate the biocompatibility of a DED Ti-coated on CoCr alloy in both in vitro and in vivo models. For viability testing, DED-Ti coated specimens showed a growth pattern similar to that of the positive control and sand-blasted specimens. No unusual values were measured, especially in the DED-Ti coated group.

In the in vivo immune profiling exam, the initial response to implantation, we observed an increased in the ratio of neutrophil within 24 h–48 h which then deceased. We also observed an increase in CD3^+^ T cells from 48 h followed by a decrease at week 2. CD4^+^ T cells were increased after 1 to 2 weeks as a delayed reaction and normalized again [40]. There were no significant differences compared to the sham group. Tissue histomorphometry analysis also identified inflammatory cells in some regions of the smooth and DED Ti-coated groups. Together, we conclude the implanted DED-Ti coated specimen does not induce a significant inflammatory response compared to the control smooth group. These results are consistent with previous studies indicating the DED Ti-surface and CoCr alloys are biocompatible for cell culture and in vivo models [14,41]. 

The reported outcomes of first-generation cementless TKA implants were poor due to limitations in the manufacturing techniques [8,9]. Since then, with the development of metal processing, dissimilar materials manufacturing, and the surface coating technology, the implant survival rate have been gradually improved and recently, better osseointegration have been reported [17,18]. 

Despite these new advances in technology, osteolysis and aseptic loosening are still a major issue. Many concerns have been raised in total hip arthroplasty (THA), which performed cementless fixation before than TKA. Wear debris generated by wear of the bearing surfaces and micro abrasion of metal component induce inflammation are considered to be the main cause for periprosthetic osteolysis. The inflammatory reaction is affected by particle characteristics, such as particle size, shape, and chemical reactivity [42,43]. Although titanium has been known to be highly biocompatible, but it has been reported to cause inflammation regardless of the particle type [43]. Smith et al. reported that Ti particles reduce mesenchymal stem cell proliferation and osteogenic differentiation [44]. The osteolysis process can be described in three stages: (1) Exaggerated inflammation induced by the activated macrophages and osteoclasts; (2) disruption of periprosthetic bone formation; and (3) disruption of bone regeneration as a consequence of the increased cytotoxic response [24]. The macrophages and neutrophils contribute to an impaired inflammatory phase. In acute phase response signaling, interleukin (IL)-6, IL-1β, prostaglandin-endoperoxide synthase (Ptgs) 2, and leukemia inhibitory factor (LIF) and ROS showed strong upregulation [25,26]. Subsequently, the GSK-3β/β-catenin signal pathway regulated osteogenic inhibition and bone destruction [27]. This induces the degradation of collagen fibers, chronic inflammation, and eventually, osteolysis. In this study, there was no significant elevation of IL-1β in the DED group compared to the sham group. The level of IL-6 increased within 24 to 48 hours after implantation which then decreased and there was no significant elevation after 4 or 8 weeks. Inflammatory cells, including neutrophil and macrophage, also increased initially after implantation which then decreased. There was no significant difference among the experimental groups.

In this regards, this newly developed DED-Ti coated process which is based on 3D metal printing technology has great advantage and can overcome the limitations of previous surface coating method by decreasing delamination of Ti-coating surfaces [14,30]. Unlike the currently used titanium plasma spray (TPS) method, DED Ti-coated method has resistant to abrasion so the risk of Ti nanoparticles formation is relatively low [29,30].

In addition, DED method is greatly beneficial in terms of mechanical stability. Unlike other technologies, DED-Ti coated process allows strong metallurgical bonding between two different materials such as Ti and CoCr alloy [30]. Also, the characteristics of the surface can be easily controlled to create ideal porous surfaces with maximum porosity, ideal pore size, and maximum roughness [20,45,46]. Therefore, DED can fabricate porous structures similar to that of human cancellous bone while maintaining the mechanical strength and this structure can improve mechanical stability. Amirhosseini et al. reported that mechanical implant instability can also induce osteolysis through similar biological and signaling pathways for aseptic loosening to titanium particles [26]. Consequently, DED is likely to reduce the risk of inflammatory pathway [47].

Our study has some limitations. First, when used as an actual TKA implant, it is in contact with osteoblast and osteoprogenitor cells. However, as the initiation of osteolysis and aseptic loosening were triggered by the inflammatory reaction, we evaluated the inflammation using fibroblast cell line which can react more sensitive to inflammatory process. Second, only cell viability and inflammatory cytokines were assessed in the in vitro study. It was adequate to evaluate the degree of inflammatory reaction, but further studies are needed to determine how they affect cell activity and proliferation. Third, tissue histomorphometry evaluation had limitations for accurate analysis because the slide was made relatively thick due to the specificity of the resin block. Furthermore, during the paraffin-slice preparation process, in some areas, the tissue was detached from the metal surface. We tried to select the most representative zone within the images in terms of tissue density and maintenance of contact surface to avoid the bias. Fourth, in this study, we compared with smooth surface and sand-blasted specimens to assess cytotoxicity only. However, further research is needed to compare DED Ti-coated with TPS and PBF, which are the current technology used in vivo model. Finally, the inflammatory reaction in human and mouse could be different, thus there are limitations in applying our results obtained from mouse model directly to humans [48,49]. Moreover, our sample size was relatively small because of experimental ethics [50]. In fact, most of the animal studies share these limitations, therefore our DED implant needs to be carefully evaluated on middle- and large-sized animal studies before clinical trial.

## 5. Conclusions

Titanium porous coating on the cobalt-chrome alloy by the 3D-DED metal printing technique does not induce chronic inflammation both in vitro and in vivo. This technology is therefore biocompatible for use in the surface coating of cementless TKA implant.
